# ATOH1/RFX1/RFX3 transcription factors facilitate the differentiation and characterisation of inner ear hair cell-like cells from patient-specific induced pluripotent stem cells harbouring A8344G mutation of mitochondrial DNA

**DOI:** 10.1038/s41419-018-0488-y

**Published:** 2018-04-19

**Authors:** Yen-Chun Chen, Chia-Ling Tsai, Yau-Huei Wei, Yu-Ting Wu, Wei-Ting Hsu, Hung-Ching Lin, Yi-Chao Hsu

**Affiliations:** 1 Institute of Biomedical Sciences, Mackay Medical College, New Taipei City, Taiwan; 2 Center for Mitochondrial Medicine and Free Radical Research Changhua Christian Hospital, Changhua, Taiwan; 3 Department of Audiology and Speech-Language Pathology, Mackay Medical College, New Taipei City, Taiwan; 4 Department of Otolaryngology, Mackay Memorial Hospital, Taipei, Taiwan

## Abstract

Degeneration or loss of inner ear hair cells (HCs) is irreversible and results in sensorineural hearing loss (SHL). Human-induced pluripotent stem cells (hiPSCs) have been employed in disease modelling and cell therapy. Here, we propose a transcription factor (TF)-driven approach using *ATOH1* and regulatory factor of x-box (*RFX*) genes to generate HC-like cells from hiPSCs. Our results suggest that *ATOH1*/*RFX1/RFX3* could significantly increase the differentiation capacity of iPSCs into *MYO7A*^mCherry^-positive cells, upregulate the mRNA expression levels of HC-related genes and promote the differentiation of HCs with more mature stereociliary bundles. To model the molecular and stereociliary structural changes involved in HC dysfunction in SHL, we further used *ATOH1/RFX1/RFX3* to differentiate HC-like cells from the iPSCs from patients with myoclonus epilepsy associated with ragged-red fibres (MERRF) syndrome, which is caused by A8344G mutation of mitochondrial DNA (mtDNA), and characterised by myoclonus epilepsy, ataxia and SHL. Compared with isogenic iPSCs, MERRF-iPSCs possessed ~42–44% mtDNA with A8344G mutation and exhibited significantly elevated reactive oxygen species (ROS) production and *CAT* gene expression. Furthermore, MERRF-iPSC-differentiated HC-like cells exhibited significantly elevated ROS levels and *MnSOD* and *CAT* gene expression. These MERRF-HCs that had more single cilia with a shorter length could be observed only by using a non-TF method, but those with fewer stereociliary bundle-like protrusions than isogenic iPSCs-differentiated-HC-like cells could be further observed using *ATOH1/RFX1/RFX3* TFs. We further analysed and compared the whole transcriptome of M1^ctrl^-HCs and M1-HCs after treatment with *ATOH1* or *ATOH1/RFX1/RFX3*. We revealed that the HC-related gene transcripts in M1^ctrl^-iPSCs had a significantly higher tendency to be activated by *ATOH1/RFX1/RFX3* than M1-iPSCs. The *ATOH1/RFX1/RFX3* TF-driven approach for the differentiation of HC-like cells from iPSCs is an efficient and promising strategy for the disease modelling of SHL and can be employed in future therapeutic strategies to treat SHL patients.

## Introduction

Degeneration or loss of inner ear hair cells (HCs) is irreversible and results in sensorineural hearing loss (SHL). In the regeneration of inner ear HCs in vitro, mouse bone marrow mesenchymal stem cells (MSCs) were the first cell type to be differentiated into HC-like cells^1^. Furthermore, mouse embryonic stem cells (ESCs) and induced pluripotent stem cells (iPSCs) have been demonstrated to be differentiated into HC-like cells^2,3^. However, it has been suggested that using chicken utricle stromal cells as feeder cells for HC differentiation may make a subsequent examination problematic^4^. Notably, Ronaghi et al.^5^ reported a feeder cell-free method for the generation of human ESC-derived HC-like cells, which exhibited many features of nascent HCs.

Proneural Atoh1 is a basic helix–loop–helix transcription factor (TF) and regulates the differentiation of HCs^6^. The ectopic expression of *Atoh1* in mouse bone marrow MSCs can result in the differentiation of HC-like cells with the expression of Myo7A and espin^1^. Atoh1 can directly transdifferentiate the supporting cells in chick cochlea to become HCs^7^. By contrast, the systemic loss of Atoh1 in mice does not result in HC differentiation^8^. However, the detection of some Myo7a and Fgf8-positive cells in *Atoh1* conditional knockout mice also suggests that the expression of these HC markers can be regulated by other factors^9^. Furthermore, the ectopic expression of *ATOH1* in human umbilical cord MSCs can lead to their differentiation to HC-like cells^10^. Notably, an increasing body of evidence has indicated that *ATOH1* gene therapy is effective for the treatment of SHL in animals^11–13^ and is under evaluation in a phase I/II clinical trial^14^. However, the *ATOH1*-mediated differentiation of HCs was still immature in vivo^15^, evidencing the need for HC differentiation.

The regulatory factor for the x-box (*RFX)* gene family has seven members in mammals (*RFX1* to *7*), with a highly conserved DNA binding domain and a winged-helix structure^16,17^ from yeast^18^, *Caenorhabditis elegans* (*daf-19*)^19^, and *Drosophila* (*dRfx*, *dRfx2*)^20^ to mammals^16^. RFX1, RFX2 and RFX3 are suggested to activate the genes in the ciliogenic pathway and mediate the transcriptional rewiring of ciliary genes^21^. Notably, *Rfx3* knockout mice demonstrated a differentiation defect in the multiciliated cells (ependymal cells) of the brain^22^. *Rfx1* and *Rfx3* conditional knockout mice are deaf due to the rapid loss of initially well-formed outer HCs (OHCs) and the deranged inner HCs (IHCs), indicating the essential roles of *Rfx1* and *Rfx3* in hearing function and the survival of terminally differentiating HCs^23^. We have previously reported that RFX1 is a negative regulator and RFX2 is a positive regulator of human *FGF1* gene activation to confer the characteristics of neural stem/progenitor cells^24–26^. In addition, RFX1, RFX2 and RFX3 can regulate *ALMS1*, which encodes a centrosomal protein and is required for the proper function of primary cilia^27^. Mutations in *ALMS1* cause Alström syndrome^28^, a disorder characterised by symptoms such as neurosensory degeneration and hearing loss^29^.

In this study, we hypothesised that ciliogenic RFX TFs may facilitate the generation of HC-like cells from human iPSCs for the disease modelling of SHL. Our findings demonstrated that *ATOH1/RFX1/RFX3* TFs could promote the differentiation of iPSC-derived HCs and facilitate the disease modelling of SHL using iPSCs from MERRF patients with A8344G mutation of mitochondrial DNA (mtDNA). The *ATOH1/RFX1/RFX3* TF-driven differentiation of HC-like cells is a promising approach for the development of future therapeutic strategies for the treatment of SHL patients.

## Results

### Differentiation of inner ear HC-like cells from hiPSCs through a non-TF method

**Fig. 1:**
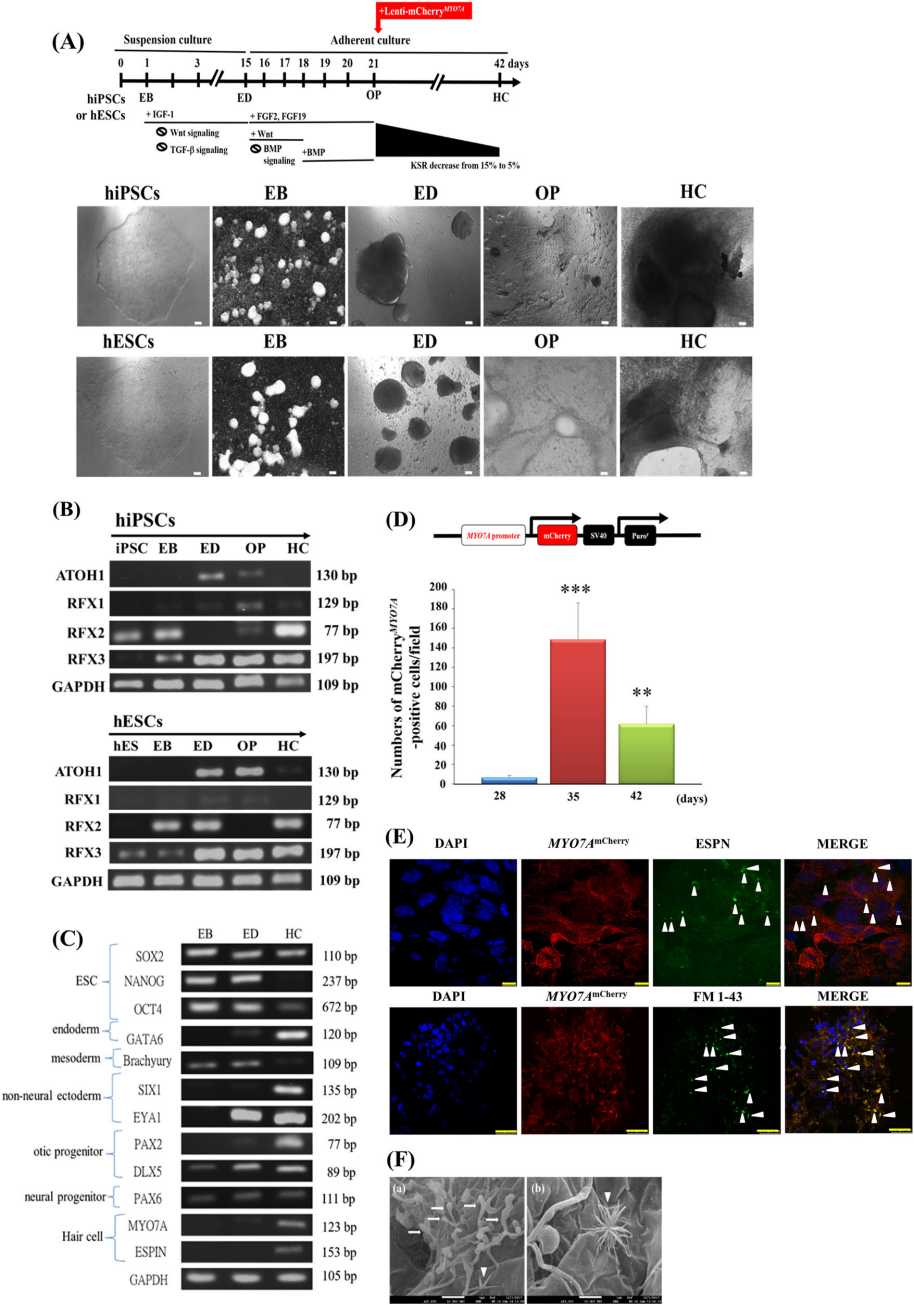
Morphological and mRNA expression analyses during hair cell (HC) differentiation from human iPSCs (hiPSCs) or human ESCs (hESCs) through a non-TF method. **a** Schematic of the 42-day otic guidance protocol. hiPSCs and hESCs exhibited a round and flat morphology in the bright field. The spherical embryoid body (EB) formation was observed on day 1. Ectoderm differentiation (ED) was observed on day 15. Otic progenitor (OP) induction was observed on day 21. The hair cell (HC) differentiation stage was observed on day 42. Scale bar = 100 µm. **b** Semiquantitative reverse transcription PCR (RT-PCR) analyses for the mRNA expression of *ATOH1*,* RFX1*, *RFX2* and *RFX3* genes at different stages (EB, ED, OP and HC) during HC differentiation from hiPSCs and hESCs. **c** ESC-specific gene transcripts (*SOX2*,* OCT4* and *NANOG*) were expressed at the EB stage. Non-neural ectoderm-specific genes (*SIX1* and *EYA1*) were expressed at the ED stage. OP-specific genes (*PAX2* and *DLX5*), the neural progenitor-specific *PAX6* gene and HC-specific genes (*MYO7A* and *ESPN*) were expressed at the HC stage. **d** mCherry^*MYO7A*^-positive cells were counted on days 28, 35 and 42. Data are presented as mean ± SD; ***p < 0.001, **p < 0.01. Positive cells were counted by randomly selecting five fields in the indicated time points. **e** Colocalisation of *MYO7A*^mCherry^ with ESPN and FM1-43-positive cells. Scale bar in ESPN staining is 10 µm, scale bar in FM 1-43 staining is 25 µm. **f** Morphology of cilia on the surface of HC-like cells, as determined through SEM. The arrow indicates the single cilium and arrowhead indicates the clustered cilia. Scale bar = 1 µm. KSR knockout serum replacement

To differentiate human inner ear HC-like cells, we initially utilised the feeder cell-free otic guidance protocol developed by Ronaghi et al.^5^ (Fig. 1a, non-TF method). Furthermore, we analysed the messenger RNA (mRNA) expression levels of *ATOH1*,* RFX1*, *RFX2* and *RFX3* during the differentiation of hiPSCs or human ESCs (hESCs) to HC (Fig. 1b) through reverse transcription PCR (RT-PCR). It has been suggested that the expression of *Atoh1* mRNA can be detected in otic progenitors (OPs) and the early immature HC stage differentiated from hESCs^5^, but not in HCs differentiated from mouse ESCs^3^. In this study, we found that the mRNA expression of *ATOH1* could be detected from the ectoderm differentiation (ED) to OP stages, but not at the late HC stage (day 42) (Fig. 1b). Furthermore, we revealed that different mRNA expression levels of *RFX1*, *RFX2* and *RFX3* were detected at the HC stage after the differentiation of hiPSCs or hESCs for 42 days. Notably, the mRNA expression levels of *RFX1* and *RFX3* could be detected at the OP stage (Fig. 1b), implying the crucial roles of *RFX1* and *RFX3* for inducing the OP into HC stage by the non-TF method.

Data from RT-PCR analyses revealed that the mRNA expression levels of pluripotency markers *SOX2*, *NANOG* and *OCT4* were downregulated during the inner ear HC differentiation process. *SOX2*, *NANOG* and *OCT4* are suggested to be crucial TFs to confer pluripotency and self-renewal ability of hESCs^30^. It has been demonstrated that *SOX2* is a marker for the prosensory identity of the otic lineage^31^. Notably, *SOX2* mRNA was continuously expressed until the HC stage (Fig. 1c). Our finding suggest that the endothermal marker *GATA6*^32^ was not expressed during embryoid body (EB) formation and ED, indicating that the mesodermal cells and endodermal cells derived from hiPSCs were suppressed by DKK-1 and SIS3. The expression of *GATA6* was significantly upregulated at the HC stage, indicating the existence of endoderm-derived cells at this stage. By contrast, Brachyury, the mesodermal marker^33^, was detected from the EB to ED stages, but not at the HC stage while using the non-TF method. The formation of the preplacodal ectoderm is crucial for cranial development. Numerous marker genes have been demonstrated to be expressed in the preplacodal ectoderm, such as *SIX1*^34^ and *EYA1*^35^. Our data indicate that the expression levels of *SIX1* and *EYA1* were upregulated during the HC differentiation process (Fig. 1c).

The transcriptional regulators *PAX2* and *DLX5* are markers of otic lineage. They have been reported to be expressed during otic differentiation from mouse^36^ and human ESCs^37^. The co-expression of these markers can serve as an indication of OP identity. *DLX5* was detectable from the EB stage to ED and HC stages. It has been revealed that the non-TF HC differentiation protocol^5^ is similar to the differentiation method of neural progenitor cells (NPCs)^38,39^. The upregulation of *PAX2* was also detected from the ED to HC stages (Fig. 1c). The mRNA expression levels of sensory inner ear HC markers, *MYO7A* and *ESPN*, were upregulated after long-term HC differentiation, indicating the formation of HC-like cells (Fig. 1c).

### Expression of HC markers in iPSC-differentiated HC-like cells through a non-TF method

The upregulation of HC markers can be used to monitor the differentiation of iPSCs to HC-like cells. We then used lentivirus carrying an *MYO7A* promoter fused with the mCherry reporter gene to infect cells at the OP stage and monitor the HC differentiation process of the hiPSCs or hESCs. The differentiated cells at the OP stage were infected with lenti-*MYO7A*^mCherry^ virus on day 21 (Fig. 1a, d). In the present study, the number of *MYO7A*^mCherry^-positive cells was semiquantitatively counted by randomly selecting five different fields in which the *MYO7A*^mCherry^–positive cells were observed. After infecting the cells at the OP stage with the lenti-*MYO7A*^mCherry^ virus on day 21, we discovered that the number of *MYO7A*^mCherry^-positive cells was significantly higher on days 35 and 42 than on day 28 (Fig. 1d). We then used immunofluorescence staining to demonstrate that *MYO7A*^mCherry^-positive cells were colocalised with the staining signal of ESPN or FM 1-43 staining (Fig. 1e). ESPN has been suggested as a critical structural marker for the actin filament cross-link in stereociliary bundles^40^. Notably, the mRNA expression levels of sensory inner ear HC markers such as *MYO7A* and *ESPN* were upregulated after long-term HC differentiation, indicating that the formation of HC-like cells occurred (Fig. 1c). In addition, it has been revealed that FM 1-43 fluorescent dye can permeate auditory HCs through ion channels. The dye rapidly and specifically labels inner ear HCs by permeating the mechanotransduction channels^40^.

### Differentiated HC-like cells failed to acquire mature stereociliary bundles through a non-TF method

We examined the morphology of the stereociliary bundle or hair bundle on the surface of HC-like cells through scanning electron microscopy (SEM). In epithelium-like areas, we observed cilium-like protrusions extending from the surface of cells. In most cases, these protrusions displayed a single cilium (the arrow in Fig. 1f) or a cluster of cilia (arrowhead in Fig. 1f); however, the clustered cilia were splayed and did not resemble the typical morphology of the mechanosensory stereociliary bundles of inner ear HCs. The lack of a typical stereociliary bundle morphology suggested that these HC-like cells may have been at a nascent state of development and may fail to completely mature in an in vitro culture system. Our observations are in agreement with those reported in studies of cultures of mouse OP^41^ and human ESCs^5^. However, the HC-like cells did not exhibit a typical mature physiological morphology, most likely due to the absence of environmental cues.

### Differentiation of HC-like cells from the iPSCs of patients with MERRF syndrome through a non-TF method

**Fig. 2:**
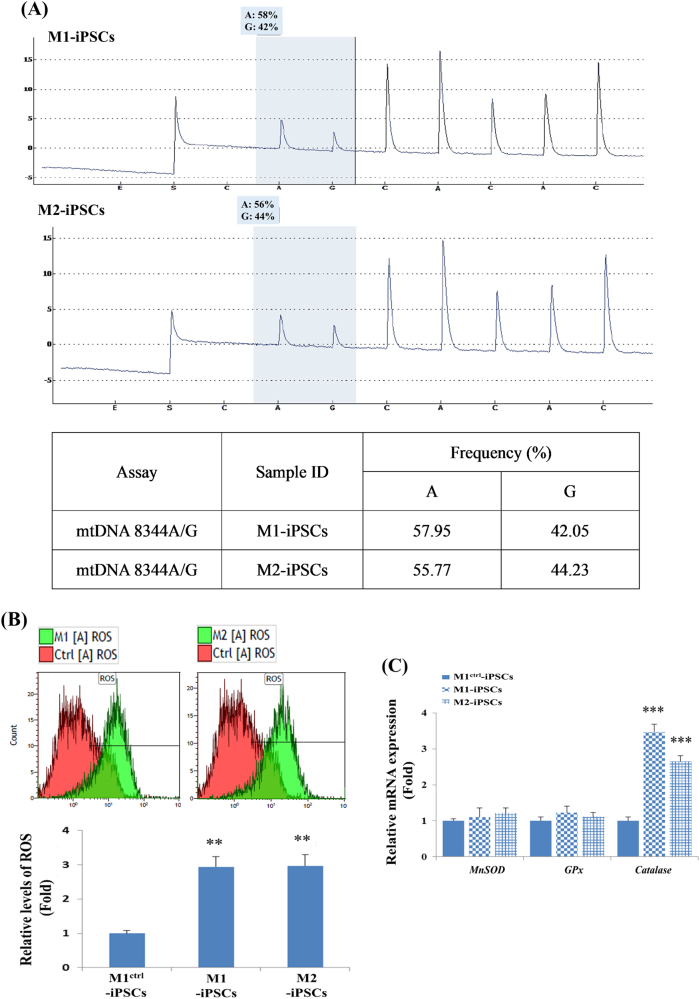
Characterisation of hiPSCs harbouring the A8344G mutation of mtDNA. **a** The proportion of mtDNA with A8344G mutation was quantified by pyrosequencing of MERRF iPSCs (M1-iPSCs and M2-iPSCs). **b** The reactive oxygen species (ROS) levels of M1-iPSCs and M2-iPSCs were higher than that of M1^Ctrl^, as indicated by flow cytometry. *p < 0.05, **p < 0.01, ***p < 0.001, *N* = 3. **c** Quantitative RT-PCR (qRT-PCR) demonstrated that the expression level of the antioxidant enzyme gene *CAT* in M1- and M2-iPSCs was significantly higher than that in M1^Ctrl^-iPSCs. *p < 0.05, **p *<* 0.01, ***p < 0.001, *N* = 3

**Fig. 3:**
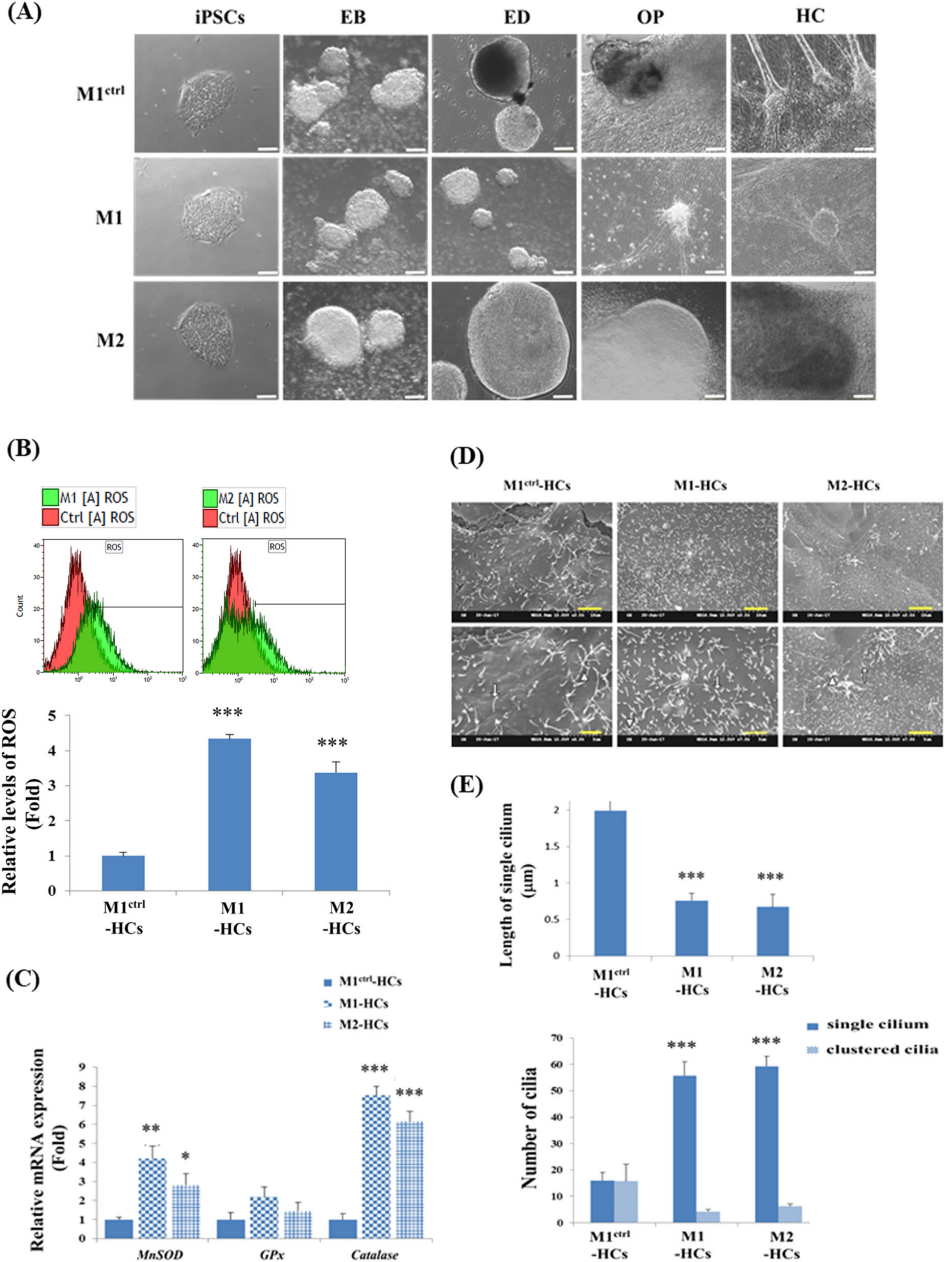
Morphological characterisation of HC-like cells from hiPSCs harbouring the A8344G mutation of mtDNA. **a** Morphology of M1^ctrl^, M1 and M2 cells during HC differentiation: these iPSCs exhibited a round and flat morphology in the bright-field imaging. The whole HC differentiation process included embryoid body (EB) formation, ectoderm differentiation (ED), otic progenitor (OP) and HC differentiation. Scale bar = 100 µm. **b** The ROS levels of M1-HCs and M2-HCs were higher than that of M1^Ctrl^-HC-like cells, as revealed by flow cytometry. *p < 0.05, **p < 0.01, ***p < 0.001, *N* *=* 3. **c** Quantitative RT-PCR (qRT-PCR) demonstrated that the expression levels of the antioxidant enzyme genes *MnSOD* and *CAT* in M1- and M2-HC-like cells were significantly higher than those in M1^Ctrl^ HC-like cells. *p < 0.05, **p < 0.01, ***p < 0.001, *N* = 3. **d** M1^Ctrl^, M1 and M2 HC-like cells obtained through a non-TF method exhibited cilia-like protrusions on the surface of cells. The arrow indicates the single cilium and arrowhead indicates the clustered cilia. Scale bar = 5 µm in the upper row of **d**, 2.5 µm in the lower row of **d**. **e** The length and number of cilia were measured from the cilia in the SEM image using ImageJ software. Data are presented as mean ± SD, *p < 0.05, **p < 0.01, ***p < 0.001., *N*=3.

MERRF syndrome is characterised by A8344G mutation of mtDNA^42^. The mutation load of A8344G mutation of mtDNA in MERRF fibroblast-derived iPSCs were determined using a pyrosequencing assay conducted at the beginning of the otic guidance differentiation. Quantitative analysis revealed the proportions of A8344G mtDNA mutation were ~42.05% in M1-iPSCs and 44.23% in M2-iPSCs (Fig. 2a). M1^ctrl^-iPSCs are isogenic iPSCs without A8344G mutation of mtDNA due to heteroplasmy during iPSC reprograming^42^. It has been revealed that the A8344G mutation of mtDNA reduced cell proliferation in hiPSCs^42^. In the present study, we observed that the A8344G mutation of mtDNA did not affect the morphology of M1-iPSC and M2-iPSC colonies during iPSC culturing (Fig. 3a).

### A8344G mutation of mtDNA causes reactive oxygen species accumulation and alters expression of antioxidant genes in iPSCs and iPSC-derived HC-like cells

To investigate the levels of ROS in iPSCs and HC-like cells derived from MERRF-iPSCs, we stained iPSCs and HC-like cells with CellROX, a green fluorescent dye used for the detection of ROSs in living cells. We revealed that the intracellular ROS levels in M1-iPSCs and M2-iPSCs were significantly higher than those in M1^Ctrl^-iPSCs (Fig. 2b). Through a non-TF differentiation method, we also discovered that the intracellular ROS levels in M1-HC-like cells and M2-HC-like cells were significantly higher than the ROS levels in M1^Ctrl^-HC-like cells (Fig. 3b). Furthermore, we analysed the expression of antioxidant genes including *MnSOD*, *GPx* and *CAT* by using quantitative RT-PCR. Notably, the results revealed a significantly increased catalase expression in M1-iPSCs and M2-iPSCs (Fig. 2c); however, their expression levels of *MnSOD* and *GPx* remained similar to those in M1^Ctrl^-iPSCs (Fig. 2c), indicating the impaired hydrogen peroxide and hydroxyl radical scavenging capacities of MERRF-iPSCs. Using a non-TF differentiation method, the significantly upregulated expression of *MnSOD* and *CAT* was observed in the M1- and M2-HC-like cells, but the expression level of *GPx* in these cells remained similar to that in the M1^Ctrl^-HC-like cells (Fig. 3c), indicating the impaired hydroxyl radical scavenging capacity of the MERRF-HC-like cells. Collectively, our results were in agreement with the findings of Chou et al.^42^, indicating elevated ROS levels and impaired ROS scavenging capacities in the MERRF-iPSCs and their differentiated progenies.

In addition, it is noteworthy that the mRNA expression levels of *ATOH1*,* RFX2 and RFX3* could be detected at the ED, OP and HC stages after the differentiation of M1 and M2-iPSCs (Supplementary Fig. 1). Notably, the mRNA expression level of *RFX1* was reduced in the OP and HC stages after the differentiation of M1 and M2-iPSCs (Supplementary Fig. 1). By contrast, the mRNA expression of *RFX1* was observed in the OP and HC stages after the differentiation of hESCs and M1^ctrl^-iPSCs (Supplementary Fig. 1), suggesting that the downregulation of mRNA expression of *RFX1* in M1-HCs and M2-HCs might also account for the defects in the stereociliary bundles of M1-HCs and M2-HCs.

### *ATOH1*, *RFX1*and* RFX3* promote the differentiation of HC-like cells and the formation of stereociliary bundles

**Fig. 4:**
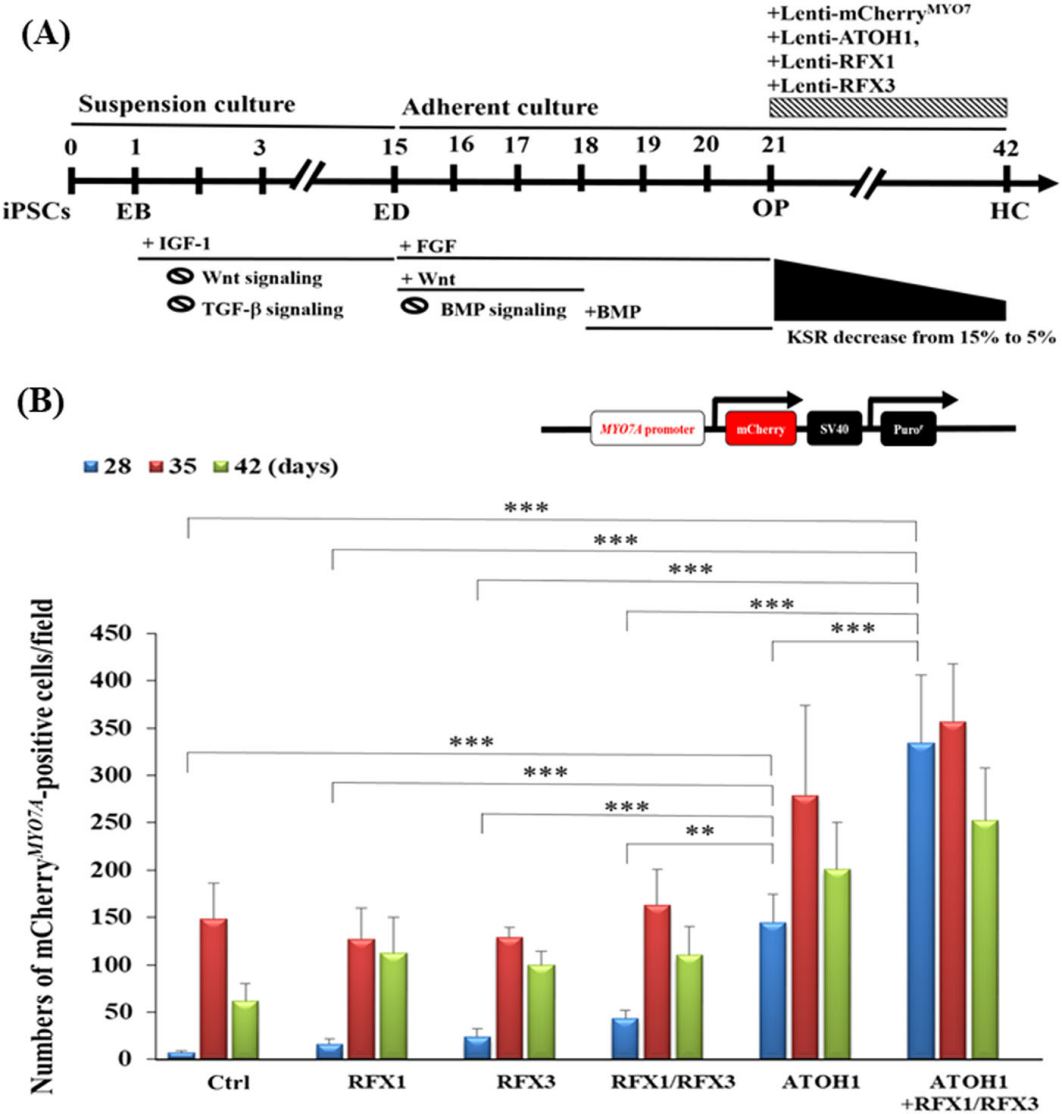
Differentiation of HC-like cells from human iPSCs (hiPSCs) through the *ATOH1/RFX1/RFX3* TF method. **a** Schematic of the HC differentiation protocol with lentiviral infections (*MYO7A*^mCherry^ reporter gene, *ATOH1*, *RFX1/RFX3* and *ATOH1/RFX1/RFX3*) on day 21. **b** The number of *MYO7A*^mCherry^-positive cells in the *ATOH1/RFX1/RFX3* and *ATOH1* conditions was higher than that in the control (Ctrl) condition. The number of *MYO7A*^mCherry^-positive cells in the *ATOH1/RFX1/RFX3* and *ATOH1* conditions was higher than those in the *RFX1/RFX3* and *ATOH1* conditions. Positive cells were counted by randomly selecting five fields in each experimental condition. Data are presented as mean ± SD, **p < 0.01, ***p < 0.001

We hypothesised that in addition to *ATOH1*, *RFX* TFs might be involved in HC differentiation. Our hypothesis was based not only on the expression profile of *RFX* TFs during the course of differentiation of iPSCs to HC (Fig. 1b), but also on observations in previous studies^23–26,43^ that have revealed that RFX1 and RFX3 TFs are essential for ciliogenesis and for the hearing function of mice. Therefore, to investigate whether *RFX1* and *RFX3* promote HC differentiation, we designed the following experiments: control (Ctrl), *ATOH1*, *RFX1*, *RFX3*, *RFX1/RFX3* and *ATOH1/RFX1/RFX3*. The *MYO7A*^mCherry^ reporter gene was used to monitor the process of HC differentiation (Fig. 4a). Notably, *ATOH1/RFX1/RFX3* efficiently promoted the differentiation of iPSCs into *MYO7A*^mCherry^-positive cells more than *ATOH1* in the early stage of HC differentiation (day 28) (Fig. 4b). Furthermore, *ATOH1* and *ATOH1/RFX1/RFX3* treatments resulted in significantly higher numbers of *MYO7A*^mCherry^-positive cells than in the Ctrl condition on day 28 (Fig. 4b); however, the *RFX1/RFX3* condition did not significantly increase the number of *MYO7A*^mCherry^-positive cells. By contrast, we found that *ATOH1* gene infection alone or in combination with *RFX1/RFX3* at the OP stage could significantly increase the number of *MYO7A*^mCherry^-positive cells on day 28, implying that the decline of *ATOH1* mRNA expression from the OP to HC stage (Fig. 1b) could be compensated by the infection of lenti-*ATOH1* at the OP stage (Fig. 4b).

**Fig. 5:**
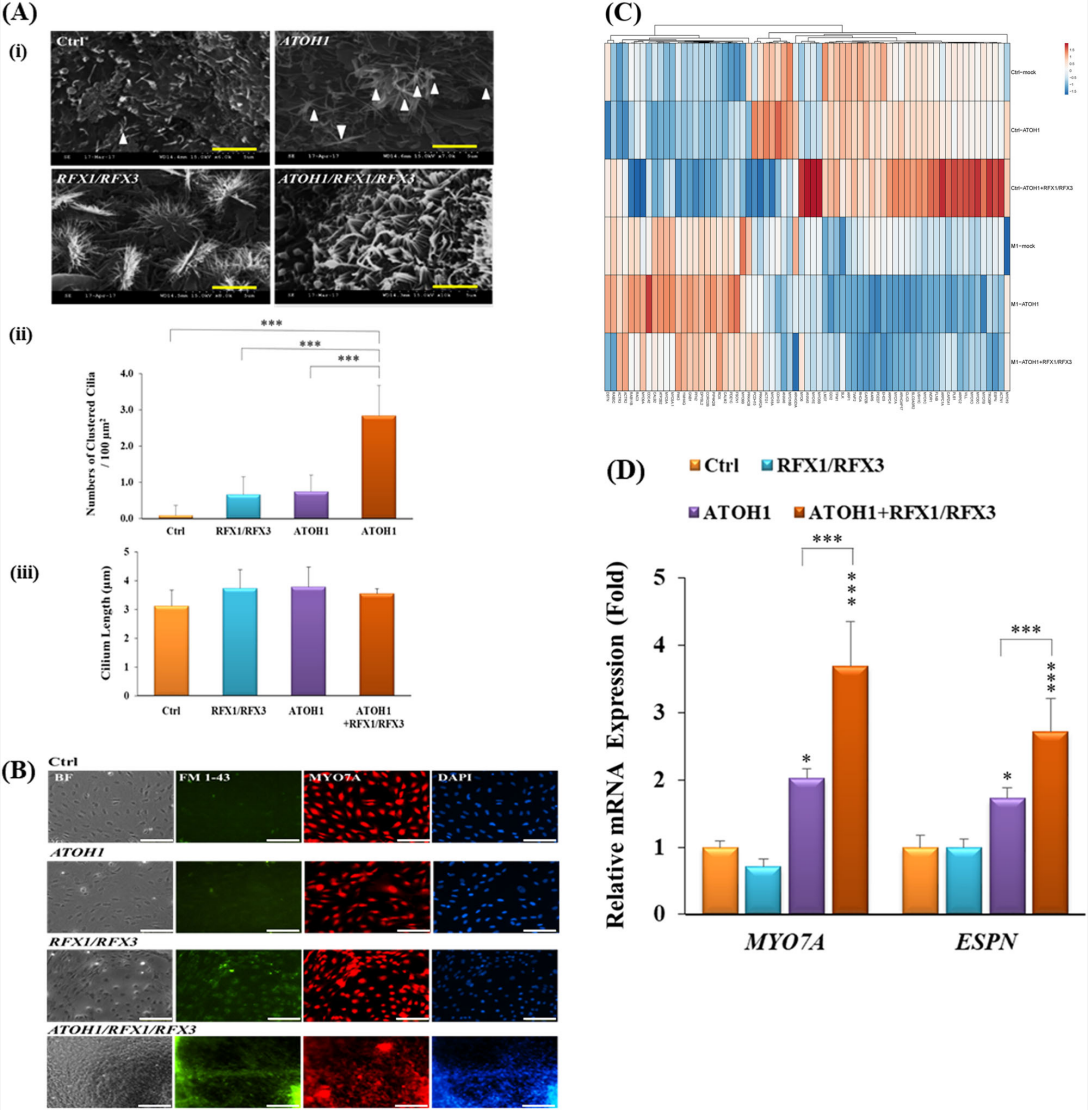
Stereociliary structure and molecular characterisation of HC-like cells differentiated from hiPSCs through the *ATOH1/RFX1/RFX3* TF differentiation method. **a**(i) HC-like cells acquired numerous cilia on the cell surfaces in the *ATOH1* condition, mostly acquired through clustered cilia in the *RFX1/RFX3* condition, and more mature stereociliary bundles were acquired in the *ATOH1/RFX1/RFX3* condition. Scale bar = 2.5 µm in the control (Ctrl), *ATOH1* and *RFX1/RFX3*, and 1.5 µm in *ATOH1/RFX1/RFX3*. (ii) The number of clustered cilia in an area of 100 μm^2^ was measured in 10 fields of the SEM image. Data are presented as mean ± SD, *p < 0.05, **p < 0.01, ***p < 0.001. (iii) Stereocilium length was measured from the cilia in 10 fields of the SEM image using ImageJ software. The average stereocilium lengths of HC-like cells in the Ctrl, *ATOH1*, *RFX1/RFX3* and *ATOH1/RFX1/RFX3* conditions were 3.52 ± 0.29, 3.41 ± 0.24 and 3.05 ± 0.23 μm, respectively. Data are presented as mean ± SD, *p < 0.05, **p < 0.01, ***p < 0.001. **b** Immunofluorescent staining for the expression of ESPN and MYO7A in the iPSC-differentiated HCs by *ATOH1*,* RFX1/RFX3* and *ATOH1/RFX1/RFX3*. **c** Whole-transcriptome analyses for the genes involved in stereociliary bundles of HC-like cells differentiated from human iPSCs through RNA sequencing. The genes involved in the characteristics of stereociliary bundles were chosen for the cluster analyses, following the procedure of Liu et al.^44^ **d** Quantitative RT-PCR analyses revealed significantly higher mRNA expression levels of *MYO7A* and *ESPN* in the *ATOH1/RFX1/RFX3* and *ATOH1* conditions than in the Ctrl condition. Furthermore, mRNA expression levels of *MYO7A* and *ESPN* in the *ATOH1/RFX1/RFX3* condition were significantly higher than those in the *ATOH1* condition. Data are presented as mean ± SD. *N* = 3, *p < 0.05, **p < 0.01, ***p < 0.001

We then investigated whether *ATOH1/RFX1/RFX3* could promote the differentiation of HC-like cells. We examined the morphology of HC-like cells derived from hiPSCs under four experimental conditions through SEM. In the Ctrl condition, we observed a few single cilia on the surface of the cells. We discovered that HC-like cells in the *ATOH1* condition harboured tightly squeezed cilia on the cell surface, and a considerable number of cilia were observed. In addition, the morphology of the cilia in HC-like cells in the *RFX1/RFX3* condition was more clustered, but the clustered cilia were splayed. In particular, the cilium morphology of HC-like cells in the *ATOH1/RFX1/RFX3* condition exhibited stereociliary bundle-like protrusions (Fig. 5a-i and Supplementary Fig. 2). The density of the clustered stereocilia in iPSC-derived HC-like cells was 0.1 ± 0.3/100 μm^2^ in the Ctrl condition, 0.7 ± 0.5/100 μm^2^ in the *RFX1/RFX3* condition, 0.8 ± 0.5/100 μm^2^ in the *ATOH1* condition and 2.8 ± 0.8/100 μm^2^ in the *ATOH1/RFX1/RFX3* condition (Fig. 5a-ii). However, the cilium lengths in each condition were similar (Fig. 5a-iii): 3.1 ± 0.5 μm in the Ctrl condition, 3.7 ± 0.7 μm in the *RFX1/RFX3* condition, 3.8 ± 0.7 μm in the *ATOH1* condition and 3.5 ± 0.2 μm in the *ATOH1/RFX1/RFX3* condition.

Furthermore, we analysed the expression of HC marker proteins including *MYO7A* and *ESPN* through immunofluorescence staining. Notably, the expression levels of ESPN in the *RFX1/RFX3* and *ATOH1/RFX1/RFX3* conditions were markedly higher than those in the Ctrl and *ATOH1* conditions, and the expression levels of *MYO7A* and *ESPN* in the *ATOH1/RFX1/RFX3* condition were higher than those in the Ctrl, *ATOH1* and *RFX1/RFX3* conditions (Fig. 5b).

### Whole-transcriptome analysis through RNA sequencing

A total of 16,272 mRNA were identified as being differentially expressed (DE) when a significance threshold of *p* ≤ 0.05 was exclusively considered. Notably, 70 of these 16,272 mRNA-encoding genes were identified as being be involved in the formation of stereociliary bundles of HCs, as suggested by Liu et al.^44^ (Fig. 5c). Our results indicate that *ATOH1/RFX1/RFX3* could enhance the mRNA expression of a cluster of genes involved in stereociliary bundles, including *ESPN* and *MYO7A* (Fig. 5c). Furthermore, we used quantitative RT-PCR to demonstrate that the expression levels of *MYO7A* and *ESPN* genes in the *ATOH1/RFX1/RFX3* condition were significantly higher than those in the Ctrl condition (Fig. 5d). Taken together, these findings suggest that *ATOH1/RFX1/RFX3* could upregulate HC marker gene expression and promote the differentiation of HC-like cells.

### Disease modelling of HC-like cells in patients with MERRF syndrome

**Fig. 6:**
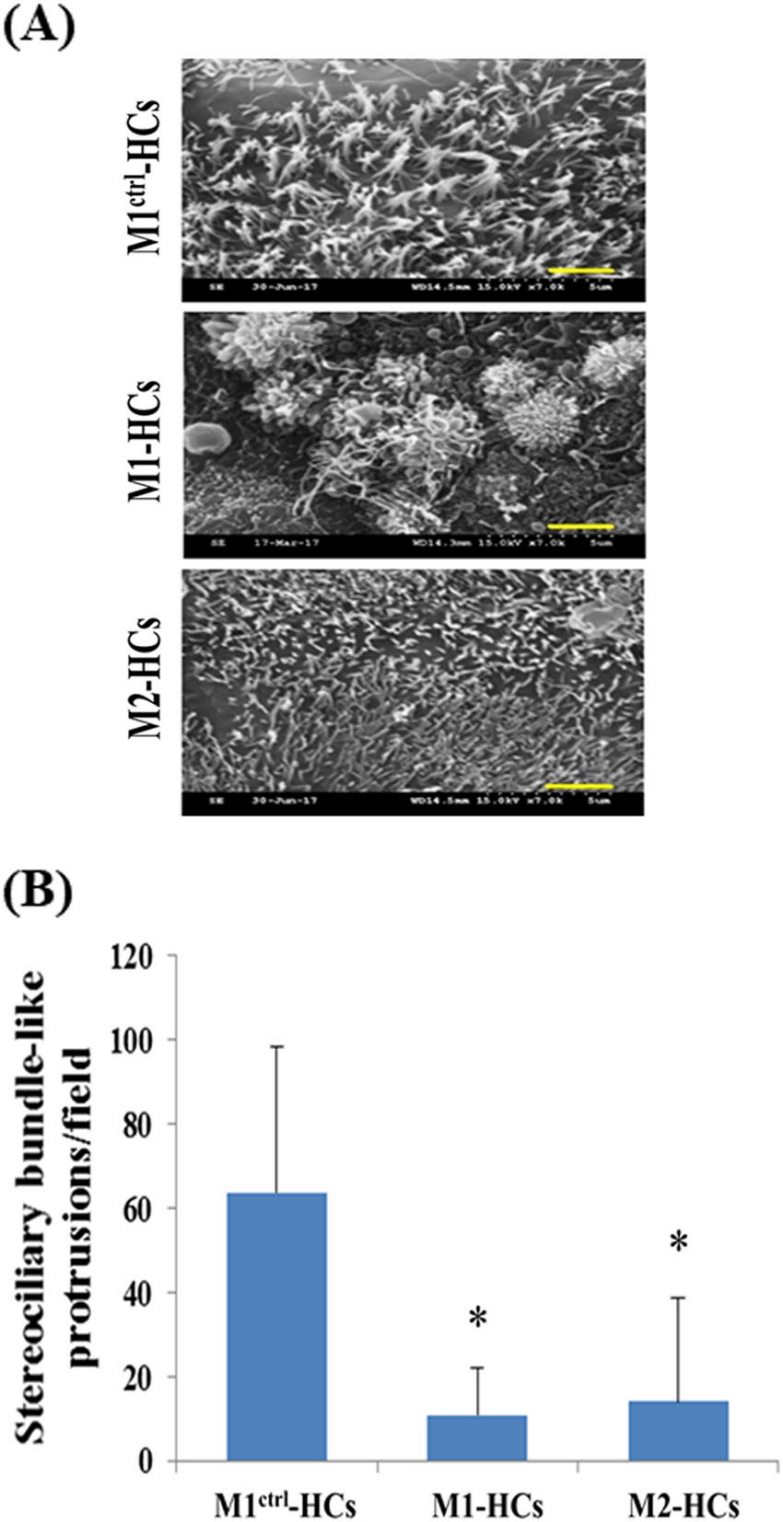
Stereociliary structure and molecular characterisation of HC-like cells differentiated from MERRF-iPSCs through an *ATOH1/RFX1/RFX3* TF-driven approach. **a** Through *ATOH1/RFX1/RFX3* TF-driven HC differentiation, M1^Ctrl^-HC-like cells acquired more mature stereociliary bundles than M1 and M2 HC-like cells. Scale bar = 2.5 µm. **b** The number of stereociliary bundle-like protrusions in a field was measured in four different fields of the SEM image. Data are presented as mean ± SD. *N* = 3, *p < 0.05

We analysed the HC differentiation capacities of M1-iPSCs and M2-iPSCs with the A8344G mutation of mtDNA. To differentiate the HC-like cells from MERRF-iPSCs, M1-iPSCs and M2-iPSCs were initially subjected to HC differentiation through a non-TF method. During the differentiation process, we observed that at the EB formation, ED induction, OP induction and HC differentiation stages, M1-iPSCs and M2-iPSCs exhibited no significant differences compared with those in M1^Ctrl^-iPSCs (Fig. 3a). The HC-like cells in the Ctrl condition also exhibited a single cilium (arrows) or a cluster of cilia (arrowheads); however, the clustered cilia were splayed and did not closely resemble the typical morphology of the stereociliary bundles of HCs (Fig. 3d). MERRF-HC-like cells that had more single cilia with shorter lengths could be observed using the non-TF method (Fig. 3e) and those with fewer stereociliary bundle-like protrusions than the control HC-like cells could be further observed using *ATOH1/RFX1/RFX3* TFs (Fig. 6a, b).

## Discussion

In this study, we developed an efficient HC differentiation protocol with *ATOH1/RFX1/RFX3* TFs by using the feeder cells-free method. The *ATOH1/RFX1/RFX3* TF-driven approach will be useful for the differentiation of HC-like cells for disease modelling and drug screening for SHL. We found that the A8344G mutation of mtDNA did not interfere with the morphology of M1- and M2-iPSCs (Fig. 3a). Furthermore, we performed RNA-sequencing experiments to analyse the whole-transcriptome levels between M1^ctrl^-HCs and M1-HCs after *ATOH1* or *ATOH1/RFX1/RFX3* treatments. Our results suggested that the HC differentiation of M1^ctrl^-iPSCs has a significantly higher tendency to be activated by *ATOH1/RFX1/RFX3* than M1-iPSCs (Fig. 5c, supplementary Figures 3 and 4). Although Chou et al.^42^ revealed that the differentiation capacities of M1-iPSCs, M2-iPSCs and isogenic M1^Ctrl^-iPSCs towards cardiomyocyte and NPCs were similar, the discrepancy in differentiation capacities among cardiomyocytes, NPCs, and HC-like cells may be due to divergent lineage differentiation protocols and the growth factors and signalling inhibitors used.

For the disease modelling of SHL, Tang et al.^45^ generated a diseased iPSC line with compound heterozygous *MYO7A* c.1184G>A and c.4118C>T mutations from deaf patients. Their results revealed that the *MYO7A* mutation did not influence the pluripotency of iPSCs. They further adopted a HC differentiation protocol for human ESCs and observed stereocilia-like protrusions after 3 weeks of culture on mitotically inactivated chicken utricle stromal cells. Although the differentiation capacities of *MYO7A*-mutation-iPSC-derived OPs were not significantly different than those of non-*MYO7A*-mutation-iPSC-derived OPs, the morphology of the stereocilia-like protrusions of the HC-like cells differentiated from *MYO7A*-mutation iPSCs was distinct from that of the protrusions in cells induced from non-*MYO7A*-mutation iPSCs. Notably, Tang et al.^45^ observed that the *MYO7A*-mutation-HC-like cells exhibited less FM1-43 uptake, abnormal electrophysiological properties and deranged stereocilia-like protrusions. These *MYO7A*-mutation-HC-like cells were curved, dishevelled and scattered, and exhibited no bonding with each other. Tang et al.^45^ corrected one of the *MYO7A*-mutation sites (c.4118C>T) in the *MYO7A*-mutation iPSCs with CRISPR/Cas9, and the HC-like cells from the corrected iPSCs exhibited a restored organisation of stereocilia-like protrusions. In addition, Chen et al.^46^ generated *MYO15A*-mutation iPSCs (c.4642G>A and c.8374G>A mutations), and demonstrated that the abnormal HC morphology, including disorganisation of F-actin bundles, abnormally short stereocilia and syncytia, and dysfunction (lower current density) of the HC-like cells from the *MYO15A*-mutation iPSCs can be corrected with CRISPR/Cas9. It is worth noting that they used the same HC differentiation protocol as Tang et al.^45^ with chicken utricle stromal cells. The stereocilium length was 3.52 ± 0.29 μm in the HC-like cells differentiated from non-*MYO15A*-mutation iPSCs, whereas the stereocilium length was 0.94 ± 0.22 μm in the HC-like cells differentiated from the *MYO15A*-mutation iPSCs. In our *ATOH1/RFX1/RFX3*-driven HC differentiation protocol, we also observed that the stereocilium length in iPSC-derived HC-like cells was 3.5 ± 0.2 μm in the *ATOH1/RFX1/RFX3* differentiation condition (Fig. 5a-iii), indicating that the stereocilium length in the HC-like cells from our *ATOH1/RFX1/RFX3*-driven HC differentiation protocol was comparable to that in the previous protocol with chicken utricle stromal cells^5^. *ATOH1/RFX1/RFX3*-differentiated HC-like cells possessed more mature stereociliary bundles than the cells obtained using the protocol involving chicken utricle stromal cells.

Interestingly, the generation of tissue-specific organoids has been suggested to be important in the modelling of cell–cell interactions in the three-dimensional level during organ development^47^. Koehler et al.^48^ reported the generation of inner ear organoids from human ESCs and iPSCs and demonstrated that inner ear organoids can have sensory epithelial cells due to the expression of HC markers, such as ESPN and MYO7A. These sensory epithelial cells are also innervated by sensory neurons. Furthermore, their electrophysiological data suggested that hESCs and hiPSC-derived organoids adopted vestibular type I and type II HCs, but not cochlear HCs. By using *ATOH1/RFX1/RFX3* TF-driven approach, the RNA-sequencing data suggested that *ATOH1/RFX1/RFX3* could significantly upregulate many genes involved in cochlear OHCs (Supplementary Figure 3) and IHCs (Supplementary Figure 4). Future studies should investigate whether the combination of *ATOH1/RFX1/RFX3* with the inner ear organoid system could facilitate the differentiation of cochlear OHCs and IHCs. In conclusion, our *ATOH1/RFX1/RFX3* TF-driven approach to generate HC-like cells from hiPSCs was efficient and promising for disease modelling. It can be employed in the development of future therapeutic strategies for the treatment of SHL patients.

## Materials and methods

### Human iPSCs and ESCs

MERRF syndrome is characterised by A8344G mutation of mtDNA, resulting in changes in the nucleotide that is normally present at the anticodon wobble nucleotide in mitochondrial tRNA^Lys^ and impairment of the synthesis of mitochondrial proteins that are fundamental for oxidative phosphorylation. Two patients, a 15-year-old Chinese girl (M1-iPSCs) and her 13-year-old sister (M2-iPSCs), were selected^42^. hiPSCs were generated from human fibroblasts derived from the patients with MERRF syndrome. MERRF-iPSCs (M1- and M2-iPSCs) were reprogrammed using four TFs, SOX2, OCT4, KLF4 and GLIS1, with retroviral vectors at Taipei Veterans General Hospital, Taiwan^42^. This study was approved by the by Institutional Review Board (IRB) of Mackay Memorial Hospital, Mackay Medical College and Taipei Veterans General Hospital. The IRB waived the informed consent requirement for the use of hiPSCs cell lines. hiPSCs were cultured on Geltrex (ThermoFisher Scientific, USA)-coated dishes in mTeSR^TM^1 medium (Stemcell Technologies, Canada). hiPSCs were generated from human fibroblasts derived from patients with MERRF syndrome. Both MERRF-iPSCs and human ESCs were generously provided by Prof. Shih-Hwa Chiou at Taipei Veterans General Hospital, Taiwan^42^. The pluripotency of M1^ctrl^-iPSCs, M1-iPSCs and M2-iPSCs was demonstrated by the expression of pluripotent markers, such as OCT4 and Nanog. The ability of M1^ctrl^-iPSCs, M1-iPSCs and M2-iPSCs to differentiate into three germ layers in vitro and form teratoma and three germ layers in vivo was also confirmed^42^. Chou et al.^42^ also characterised the expression of these markers in iPSCs-derived cardiomyocyte and NPCs in their publication. Notably, changes such as the elevation of ROS levels and impaired antioxidant *catalase* gene expression in M1^ctrl^-iPSCs/M1-iPSCs/M2-iPSCs and M1^ctrl^-HCs/M1-HCs/M2-HCs (Figs. 2 and 3) were similar to the previous findings in cardiomyocytes and NPCs differentiated from M1^ctrl^ -iPSCs, M1-iPSCs and M2-iPSCs^42,57^.

### HC differentiation from iPSCs

To differentiate human inner ear HC-like cells, we initially utilised the feeder cell-free otic guidance protocol developed by Ronaghi et al.^5^ (non-TF method). First, we generated EBs from hiPSCs or hESCs through a suspension cell culture with the addition of insulin growth factor-1 (IGF-1), which is an inhibitor of WNT and TGF-β signalling (Fig. 1a). Following EB formation, the EBs were treated with the inhibitors of TGF-β and WNT signalling pathways to suppress the differentiation of endodermal and mesodermal lineages and promote cells towards the formation of the cranial ectoderm^3^. The downregulation of WNT and TGF-β signalling can result in the formation of a primitive streak by the treatment of DKK-1 (Wnt inhibitor) and the selective inhibitor of Smad3 (SIS3, interference with TGF-β signalling), thus increasing the ED during EB formation. In addition, it has been revealed that IGF-1 and IGF signalling are crucial for the differentiation of the cranial ectoderm^49^. After treatment of EBs with IGF-1, DKK-1 and SIS3, the otic induction phase was initiated in an adherent cell culture. These EBs were then treated to induce the activation of the FGF signalling pathway, which was followed by an initial period of BMP inhibition with noggin, and then WNT activation with R-spondin 1. These EBs were then differentiated to the stage of presumptive OPs. It has been demonstrated that OPs independently differentiate into the surrounding tissues and do not require external signalling for correct differentiation^50,51^. Therefore, a long-term culture with a decreasing concentration of knockout serum replacement (KSR) was used in this study for the differentiation of HC-like cells (Fig. 1a)^57^.

### Embryoid body formation

For EB formation, hiPSCs or hESCs were dissociated with dispase (Stemcell Technologies) and transferred to ultralow attachment surface plates (Corning, USA) containing the mTeSR™1 medium. EBs were cultured in ultralow attachment surface plates containing mTeSR™1 medium supplemented with 100 ng/mL of recombinant human DKK-1 (R&D Systems, USA), 3 μM of SIS3 and 10 ng/mL of IGF1 (both were from Sigma-Aldrich Chemical Co., USA) for 15 days^5,57^.

### Induction of otic progenitors

For OP induction, the EBs were transferred into a plate coated with a poly-l-ornithine and laminin (both were from Sigma-Aldrich Chemical Co.)-coated plate and cultured for 3 days in an advanced Dulbecco’s modified Eagle’s medium (DMEM)/nutrient mixture F12 supplemented with 20% KSR, N2, B27 (ThermoFisher Scientific), human basic fibroblast growth factor (bFGF; 25 ng/mL; R&D Systems), human FGF19 (25 ng/mL; R&D Systems), human noggin (30 ng/mL; R&D Systems), human R-spondin1 (R&D Systems; 50 ng/mL), heparan sulphate (50 ng/mL; Sigma, USA) and ampicillin (50 µg/mL). However, the medium was replaced with the advanced DMEM/F12 supplemented with 15% KSR, N2, B27, bFGF (25 ng/mL), human FGF19 (25 ng/mL), human BMP4 (20 ng/mL; R&D Systems), heparan sulphate (50 ng/mL) and ampicillin (50 µg/mL) and cultured for 3 days^5,57^.

### Inner ear HC-like cells differentiation

For inner ear HC-like cell differentiation, the medium was replaced with the advanced DMEM/F12 supplemented with 15% KSR, N2 and B27, and ampicillin (50 mg/mL). The concentration of KSR progressively decreased until day 42. We used the lentivirus vector pEZX-LvPM02 (GeneCopoeia, Rockville, MD, USA) that carried the *MYO7A* promoter-driven mCherry as a reporter gene to infect differentiated cells on day 21 of the OP stage for the monitoring of the HC differentiation^5,57^. The lentiviral construct of *MYO7A*^mCherry^ reporter used in this study was purchased from GeneCopoeia™ (HPRM25722-PG02, GeneCopoeia). The promoter details are as follows: >HPRM25722, NM_000260, NM_001127179; Entrez_ID = 4647; chr11+:76837906-76839534; −1404 to +224, length = 1629. We followed the method that Boëda et al.^52^ previously reported in the generation of human *MYO7A* promoter-GFP transgenic mice for targeting the *MYO7A*-positive cells in vivo. The expression of the GFP reporter gene was under the control of the human *MYO7A* promoter region −2109 to +2370. Notably, the promoter region (−1404 to +224) of the *MYO7A*^mCherry^ reporter gene used in our study was within the *MYO7A* promoter (−2109 to +2370) in *MYO7A* promoter-GFP transgenic mice. These GFP transgenic mice were found to have the following characteristics: (1) GFP expression was specifically restricted to HCs in the inner ear and cochlear and (2) GFP expression was not observed in other organs^52^. To confirm the specificity of the *MYO7A*^mCherry^ reporter gene, we used immunofluorescence double-staining experiments to demonstrate the colocalisation of *MYO7A*^mCherry^ signal with mature HC markers (ESPN and FM1-43) (Fig. 1e). We attempted to dissociate the HC-like cells from human iPSCs and quantify the mCherry-positive cells through flow cytometry after HC differentiation. However, the HC-like cells formed a tissue-like structure and were difficult to dissociate into single cells to quantify the percentage of *MYO7A*^mCherry^-positive cells through flow cytometry. We therefore chose to semiquantitatively count the number of *MYO7A*^mCherry^-positive cells in the tissue-culture wells of iPSC-derived HC-like cells under a florescent microscope (Fig. 1d, e). Consistently, the number of *MYO7A*^mCherry^-positive cells was positively correlated with the mRNA expression of *MYO7A* in the HC stage at day 42 after differentiation (Fig. 1c, d).

### Production of viruses

Lentiviral vectors lenti-RFX1 and lenti-RFX3 composed from the pSG5-RFX1 and pSG5-RFX3 constructs were provided by Shaul^53^ and Iwama^54^. The lenti-ATOH1 vector was purchased from GeneCopoeia. Lenti-ATOH1, lenti-RFX1, lenti-RFX3 and lenti- *MYO7A*^mCherry^ vectors were transfected into HEK293T cells. After 48 h, viruses were collected. Viral supernatants were concentrated using an ultracentrifuge for 2 h at 100,000×*g*. A Global UltraRapid Lentiviral Titer Kit (System Biosciences Inc., Mountain View, CA, USA) was used to determined viral titres^57^.

### Reverse transcription PCR

Total RNA was isolated using FavorPrep Tissue Total RNA Extraction Mini Kit (Favorgen, Ping-Tung, Taiwan). Afterwards, Tri-Reagent RNA clean-up kit (Favorgen, Ping-Tung, Taiwan) was applied, and complementary DNA (cDNA) was synthesised using the iScript cDNA synthesis kit (Bio-Rad, USA). A PCR was performed using the KAPA2G ReadyMix PCR kit (Kapa Biosystems, Cape Town, South Africa)^57^. The gene-specific primer sequences are presented in Supplementary Table 1.

### RNA isolation and library construction and sequencing

The total RNA samples were first treated with DNase I to degrade any possible DNA contamination. Then, the mRNA was then enriched using the oligo (dT) magnetic beads. Mixed with the fragmentation buffer, the mRNA was fragmented into short fragments. Then, the first strand of cDNA was synthesised using random hexamer primer. Buffer, dNTPs, RNase H and DNA polymerase I were added to synthesise the second strand. The double-strand cDNA was purified with magnetic beads. End reparation and 3′-end single-nucleotide A (adenine) addition was then performed. Finally, sequencing adaptors were ligated to the fragments. The fragments were enriched through PCR amplification. During the quality control (QC) step, Agilent 2100 Bioanaylzer and ABI StepOnePlus Real-Time PCR System were used to qualify and quantify the sample library. The library products were then ready for sequencing through Illumina HiSeq TM 2000 or other sequencers. The RNA-sequencing experiments were performed by Genomics Inc. (New Taipei City, Taiwan).

### Bioinformatic analyses

Primary sequencing data produced by Illumina Nextseq TM 500, called raw reads, was subjected to QC to determine if a resequencing step is was required. After QC, raw reads were filtered into clean reads, which are for alignment to the reference sequences. A QC of the alignment was performed to determine if resequencing was required. The alignment data was utilised to calculate the distribution of reads on reference genes and the mapping ratio. If alignment result passed the QC, we proceeded with the downstream analysis including gene expression and a deep analysis based on gene expression (e.g., principal component analysis (PCA)/correlation/screening DE genes). The bioinformatic analyses were performed by Genomics Inc.

### Quantitative PCR

Total RNA was isolated using the FavorPrep RNA clean-up kit (Favorgen), and cDNA was synthesised using the iScript cDNA synthesis kit (Bio-Rad) according to the manufacturer’s instructions. PCR was performed using the KAPA SYBR FAST qPCR kit (Kapa Biosystems) and the 7900HT fast real-time PCR system^57^. The gene-specific primer sequence is presented in Supplementary Table 1.

### Immunofluorescent staining

The cells were fixed with 4% paraformaldehyde for 15 min at room temperature. Nonspecific binding sites were blocked, and an additional permeabilisation step was performed for 1 h in 0.2% Triton X-100 and 1% bovine serum albumin. The cells were incubated overnight with a primary antibody at 4 °C. Following this incubation, the fluorescein isothiocyanate- and tetramethylrhodamine-conjugated species secondary antibodies (1:500, Invitrogen, Carlsbad, CA, USA) were used to detect the primary antibodies. The primary antibodies used in this study were anti-MyosinVIIa (1:500, Abcam, Cambridge, MA, USA) and anti-Espin (1:500, Abcam). Nuclei were visualised using the Hoechst stain (Invitrogen). For FM1-43 staining (Invitrogen), the cells were immersed in the stain solution on ice for 1 min according to the manufacturer’s instructions. We used FM 1-43 staining to demonstrate that the iPSC-derived differentiated HC-like cells exhibit the expression of not only HC markers (MYO7A and ESPN) but also specific ion channels in HCs^57^. It was reported that FM 1-43 can be used to characterise HCs^55^ and examine the recycling of synaptic vesicle-associated activities^56^.

### Scanning electron microscopy

The cells were fixed with 4% glutaraldehyde for 1 h, dehydrated in a graded ethanol series and dried using critical point drying with liquid CO_2_. Specimens were sputter-coated with 100 Å Au/Pd and viewed using a Hitachi S-3500N variable pressure SEM operated under a high vacuum of 5–10 kV. The SEM experiments were performed in the Core Facility of SEM in Mackay Memorial Hospital^57^.

### Statistics analyses

Data are expressed as means ± SD. Independent *t* test was used for comparison of two groups. One-way analysis of variance was used for comparison of multi-groups. The difference of data was considered statistically significant at *p* < 0.05.

## Electronic supplementary material

Supplementary table 1

Supplementary Figure 1

Supplementary Figure 2

Supplementary Figure 3

Supplementary Figure 4
