# Supplementary material for: ATOH1/RFX1/RFX3 transcription factors facilitate the differentiation and characterisation of inner ear hair cell-like cells from patient-specific induced pluripotent stem cells harbouring A8344G mutation of mitochondrial DNA
Source: Cell Death Dis. 2018 Apr 19;9(4):437. doi: 10.1038/s41419-018-0488-y (PMC5941227; doi:10.1038/s41419-018-0488-y)
Supplement: Supplementary file 1 — Supplementary table 1 [file 41419_2018_488_MOESM1_ESM.docx]

**Supplementary table 1. Primer sets for PCR and qPCR**

| **Primer** | **Sequence** | **Product Size (bp)** |
| --- | --- | --- |
| SOX2-F | 5’-TACAGCATGTCCTACTCGCAG-3’ | 110 |
| SOX2-R | 5’-GAGGAAGAGGTAACCACAGGG-3’ |  |
| OCT4-F | 5’-CTTCAGGCACTGTGTTCATTG-3’ | 672 |
| OCT4-R | 5’-TTTGGCTGAACACCTTCCCA-3’ |  |
| NANOG-F | 5’-AAGGTCCCGGTCAAGAAACAG-3’ | 237 |
| NANOG-R | 5’-CTTCTGCGTCACACCATTGC-3’ |  |
| GATA6-F | 5’-CTCAGTTCCTACGCTTCGCAT-3’ | 120 |
| GATA6-R | 5’-GTCGAGGTCAGTGAACAGCA-3’ |  |
| Brachyury-F | 5’-TATGAGCCTCGAATCCACATAGT-3’ | 109 |
| Brachyury-R | 5’-CCTCGTTCTGATAAGCAGTCAC-3’ |  |
| SIX1-F | 5’-CTGCCGTCGTTTGGCTTTAC-3’ | 135 |
| SIX1-R | 5’-GCTCTCGTTCTTGTGCAGGT-3’ |  |
| EYA1-F | 5’-GTCACAGTCTCAGTCACCTGG-3’ | 202 |
| EYA1-R | 5’-GGGATAAGACGGATAGTCCTGC-3’ |  |
| PAX2-F | 5’-CGGCTGTGTCAGCAAAATCC-3’ | 77 |
| PAX2-R | 5’-GCTTGGAGCCACCGATCA-3’ |  |
| PAX6-F | 5’-TGGGCAGGTATTACGAGACTG-3’ | 111 |
| PAX6-R | 5’-ACTCCCGCTTATACTGGGCTA-3’ |  |
| DLX5-F | 5’-GTCTTCAGCTACCGATTCTGAC-3’ | 89 |
| DLX5-R | 5’-CTTTGCCATAGGAAGCCGAG-3’ |  |
| MYO7A-F | 5’-GCAGAACGCAACGCACATC-3’ | 123 |
| MYO7A-R | 5’-TCCCGGTAGCGGATAAGCA-3’ |  |
| ATOH1-F | 5’-CCTTCCAGCAAACAGGTGAAT-3’ | 130 |
| ATOH1-R | 5’-TTGTTGAACGACGGGATAACAT-3’ |  |
| RFX1-F | 5’-AGACCGGCGTTCCTACTCA-3’ | 129 |
| RFX1-R | 5’-GCAGCGTAGTGGATAGGCAG-3’ |  |
| RFX2-F | 5’-GCGATTGAA AACCTCCAAAA-3’ | 77 |
| RFX2-R | 5’-GGCTTCAGACGAATCCCATA-3’ |  |
| RFX3-F | 5’-AAACTGGACCCAGTCAATGC-3’ | 197 |
| RFX3-R | 5’-TGTTGCATGGGTTGTTGTCT-3’ |  |
| ESPN-F | 5’-CAGAGTGCAGGACAAAGACAA-3’ | 153 |
| ESPN-R | 5’-GCAGCGTAGTGGATAGGCAG-3’ |  |
| GAPDH-F | 5’-TGGTGGCAGTTACCTTACTACT-3’ | 105 |
| GAPDH-R | 5’-CAAGGGCTCTTGATTTGCTGA-3’ |  |
