# Supplementary figures and images for: ATOH1/RFX1/RFX3 transcription factors facilitate the differentiation and characterisation of inner ear hair cell-like cells from patient-specific induced pluripotent stem cells harbouring A8344G mutation of mitochondrial DNA
Source: Cell Death Dis. 2018 Apr 19;9(4):437. doi: 10.1038/s41419-018-0488-y (PMC5941227; doi:10.1038/s41419-018-0488-y)

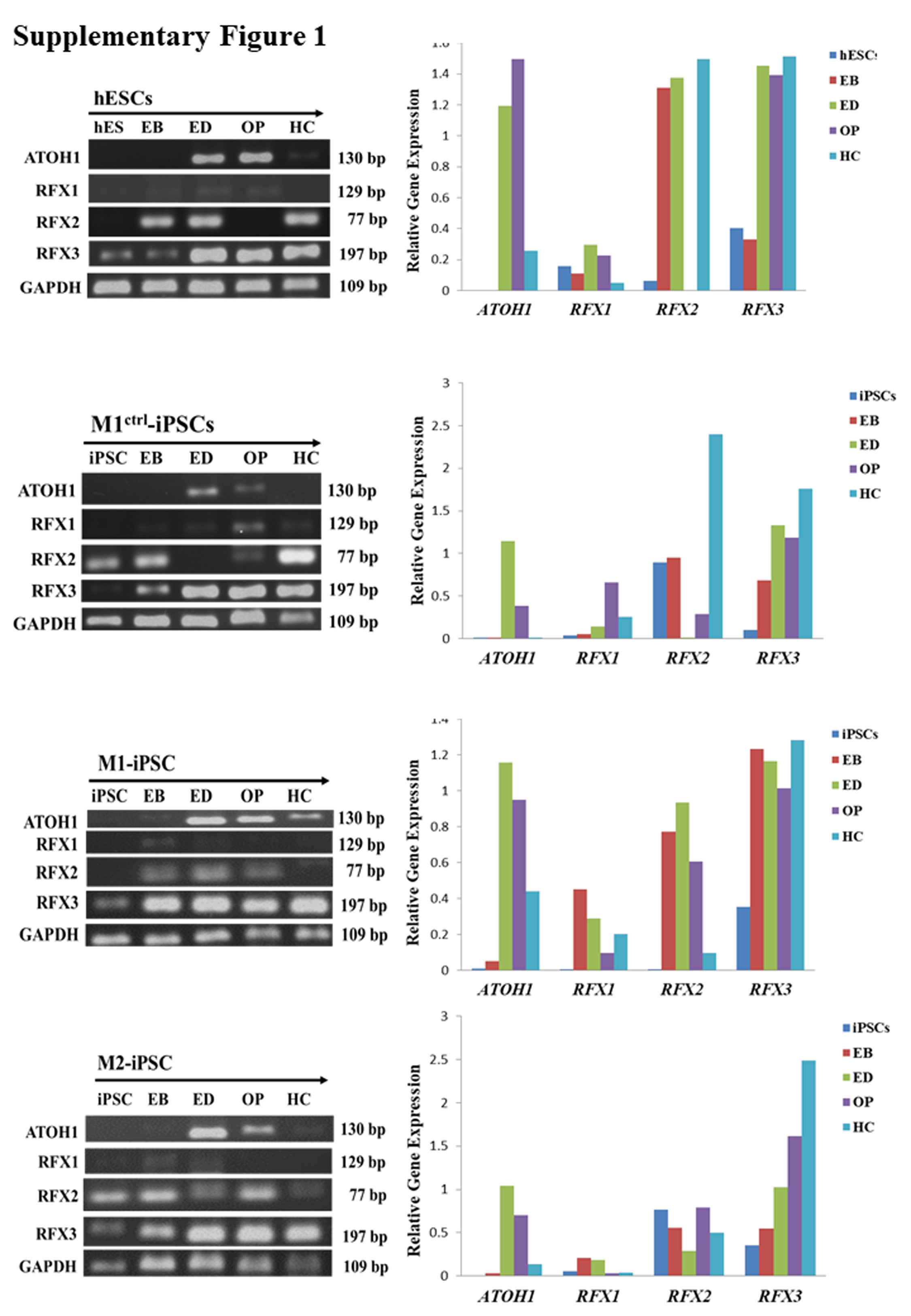

Supplement: Supplementary file 2 — Supplementary Figure 1 [file 41419_2018_488_MOESM2_ESM.tif]

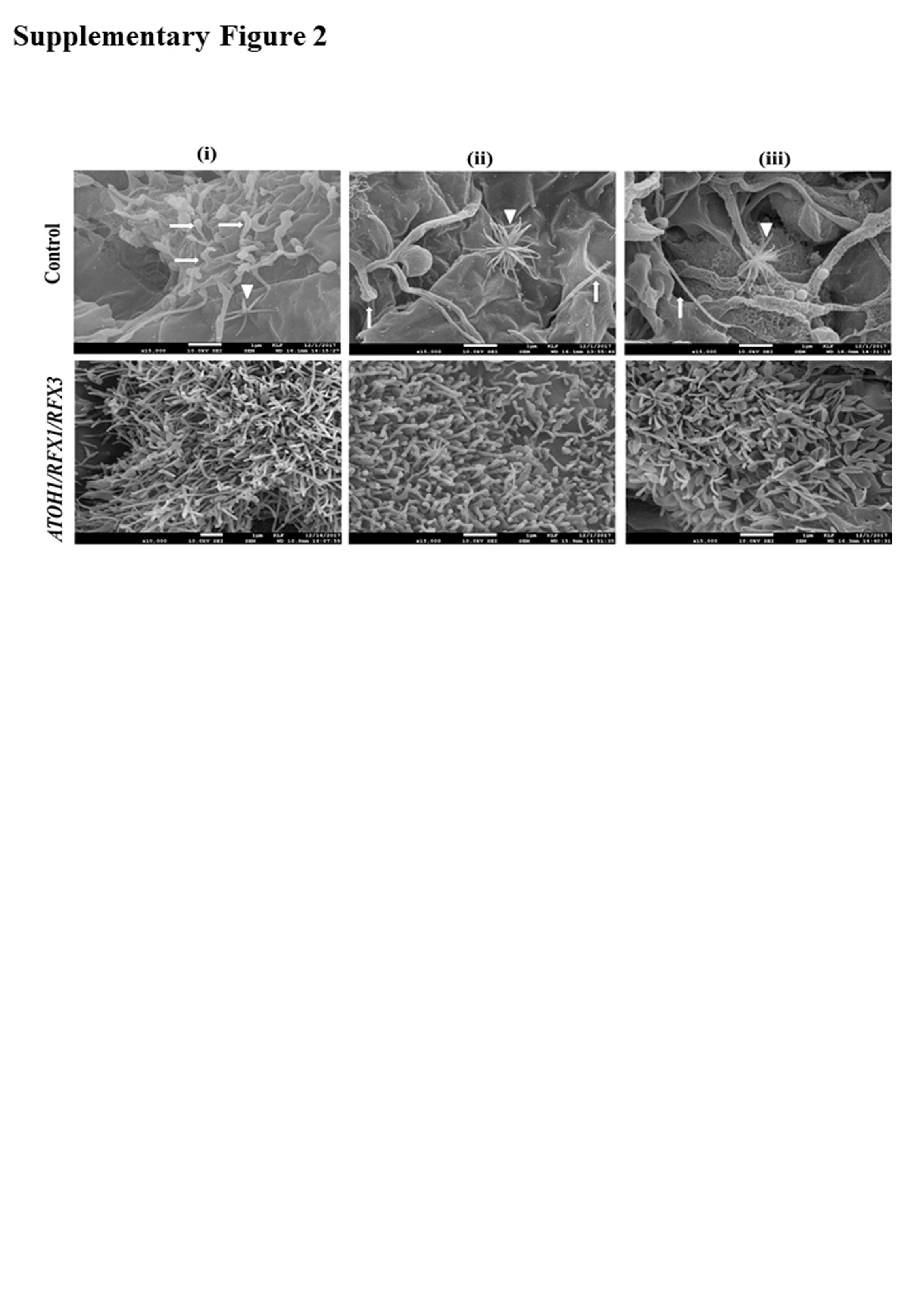

Supplement: Supplementary file 3 — Supplementary Figure 2 [file 41419_2018_488_MOESM3_ESM.tif]

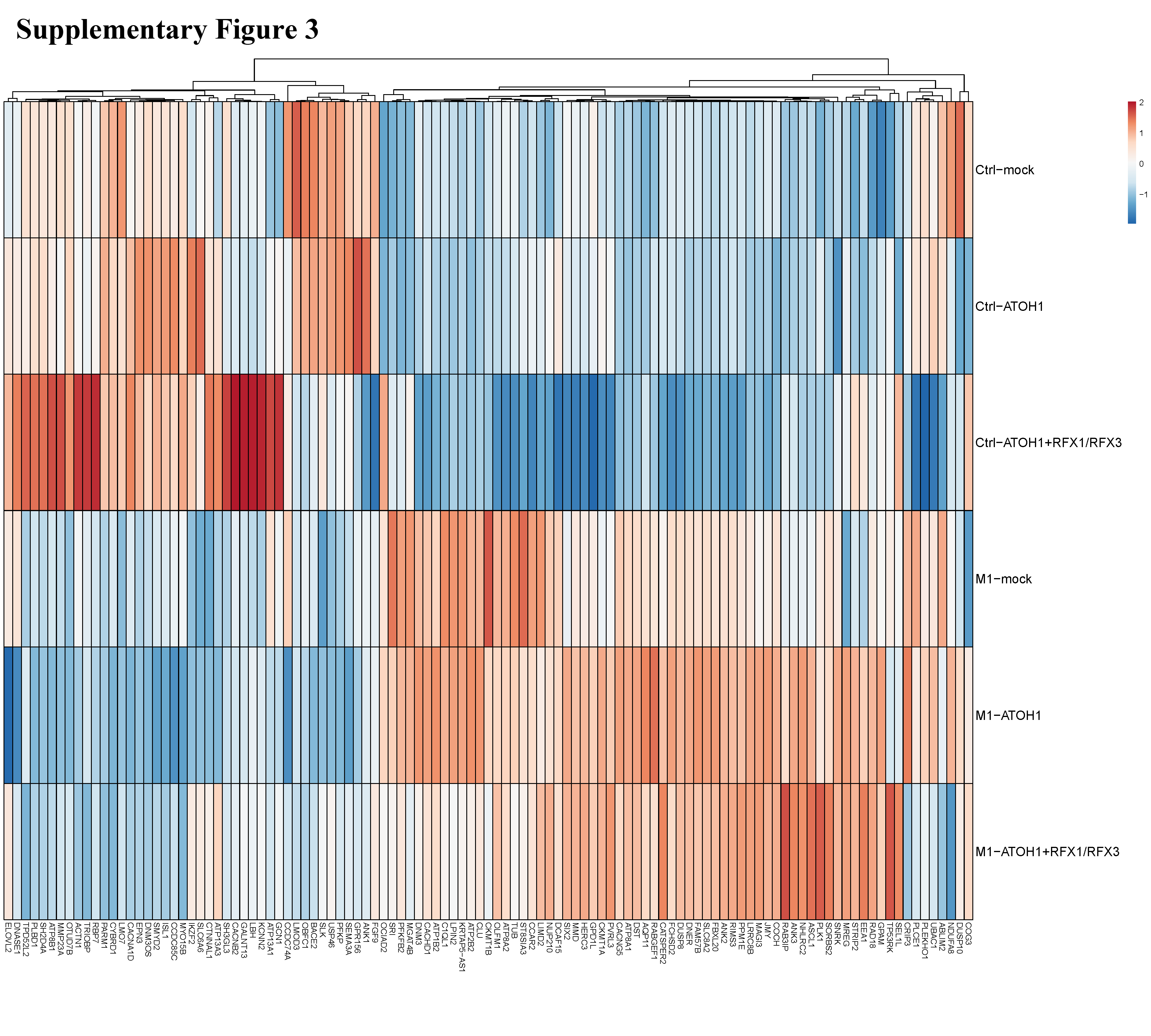

Supplement: Supplementary file 4 — Supplementary Figure 3 [file 41419_2018_488_MOESM4_ESM.tif]

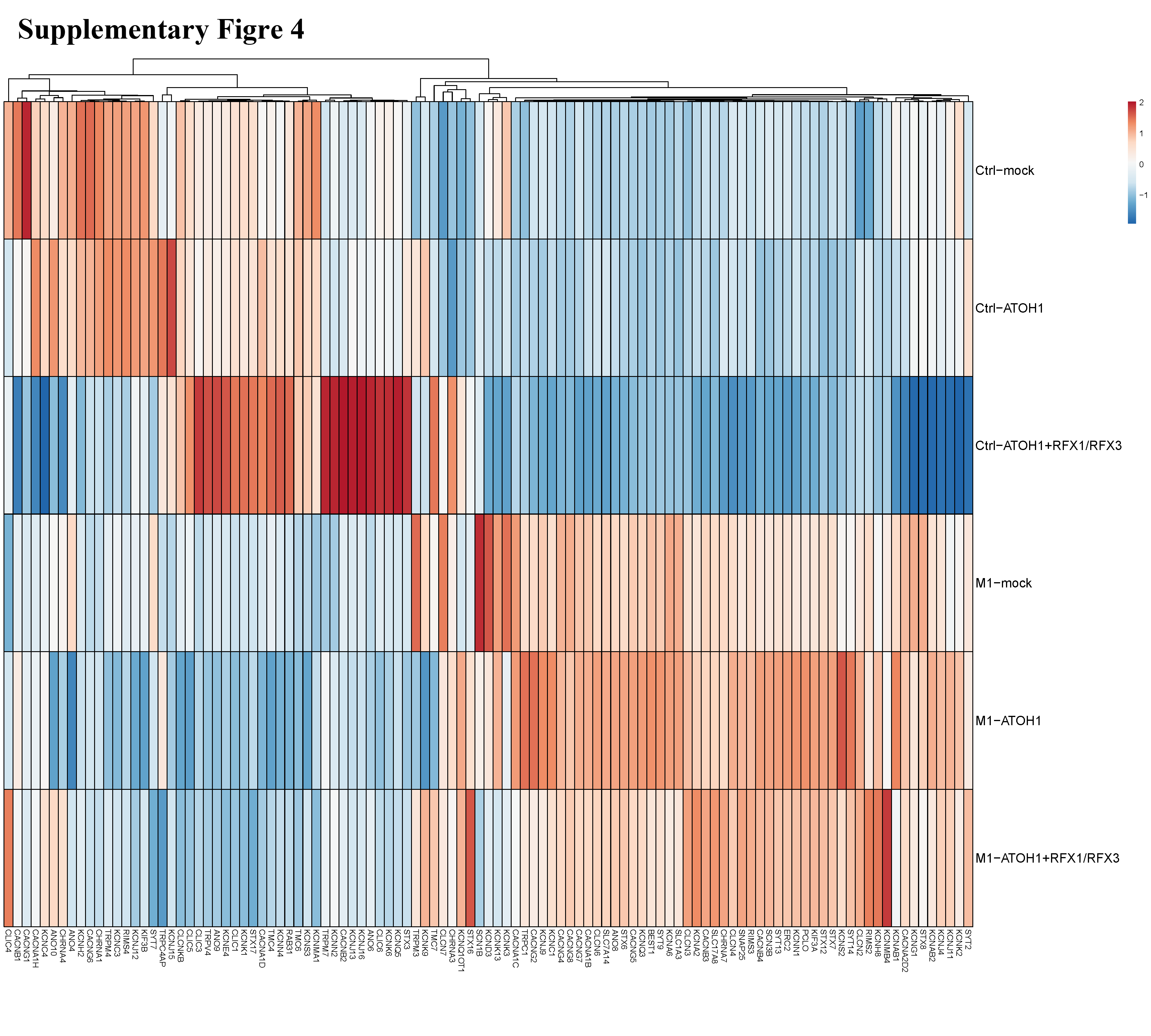

Supplement: Supplementary file 5 — Supplementary Figure 4 [file 41419_2018_488_MOESM5_ESM.tif]
